# Expression of Concern: A Dynamic Adaptive Ensemble Learning Framework for Noninvasive Mild Cognitive Impairment Detection: Development and Validation Study

**DOI:** 10.2196/75352

**Published:** 2025-04-04

The publisher expresses concern regarding the following article:

A Dynamic Adaptive Ensemble Learning Framework for Noninvasive Mild Cognitive Impairment Detection: Development and Validation Study [1].

This article is under investigation for potential peer review irregularities. Readers are advised to interpret the findings with caution pending the outcome of this inquiry.

## References

[1] Li A, Li J, Hu Y, Geng Y, Qiang Y, Zhao J (2025). A Dynamic Adaptive Ensemble Learning Framework for Noninvasive Mild Cognitive Impairment Detection: Development and Validation Study. JMIR Med Inform.

